# Differential characteristics of bipolar I and II disorders: a retrospective, cross-sectional evaluation of clinical features, illness course, and response to treatment

**DOI:** 10.1186/s40345-023-00304-9

**Published:** 2023-07-14

**Authors:** Giulio Emilio Brancati, Abraham Nunes, Katie Scott, Claire O’Donovan, Pablo Cervantes, Paul Grof, Martin Alda

**Affiliations:** 1 Psychiatry Unit 2, Department of Clinical and Experimental Medicine, University Hospital of Pisa, Pisa, Italy; 2 Department of Psychiatry, QEII Health Sciences Centre, Dalhousie University, 5909 Veterans’ Memorial Lane, Abbie J. Lane Memorial Building (room 3088), Halifax, NS B3H 2E2 Canada; 3 Faculty of Computer Science, Dalhousie University, Halifax, NS Canada; 4 Department of Psychiatry, McGill University Health Centre, Montreal, QC Canada; 5 Mood Disorders Center of Ottawa, Ottawa, ON Canada; 6 Department of Psychiatry, University of Toronto, Toronto, ON Canada

**Keywords:** Bipolar disorder, Bipolar disorder type I, Bipolar disorder type II, Age at onset, Family history, Clinical course, Predominant polarity, Rapid cycling, Suicide, Lithium response

## Abstract

**Background:**

The distinction between bipolar I and bipolar II disorder and its treatment implications have been a matter of ongoing debate. The aim of this study was to examine differences between patients with bipolar I and II disorders with particular emphasis on the early phases of the disorders.

**Methods:**

808 subjects diagnosed with bipolar I (N = 587) or bipolar II disorder (N = 221) according to DSM-IV criteria were recruited between April 1994 and March 2022 from tertiary-level mood disorder clinics. Sociodemographic and clinical variables concerning psychiatric and medical comorbidities, family history, illness course, suicidal behavior, and response to treatment were compared between the bipolar disorder types.

**Results:**

Bipolar II disorder patients were more frequently women, older, married or widowed. Bipolar II disorder was associated with later “bipolar” presentation, higher age at first (hypo)mania and treatment, less frequent referral after a single episode, and more episodes before lithium treatment. A higher proportion of first-degree relatives of bipolar II patients were affected by major depression and anxiety disorders. The course of bipolar II disorder was typically characterized by depressive onset, early depressive episodes, multiple depressive recurrences, and depressive predominant polarity; less often by (hypo)mania or (hypo)mania-depression cycles at onset or during the early course. The lifetime clinical course was more frequently rated as chronic fluctuating than episodic. More patients with bipolar II disorder had a history of rapid cycling and/or high number of episodes. Mood stabilizers and antipsychotics were prescribed less frequently during the early course of bipolar II disorder, while antidepressants were more common. We found no differences in global functioning, lifetime suicide attempts, family history of suicide, age at onset of mood disorders and depressive episodes, and lithium response.

**Conclusions:**

Differences between bipolar I and II disorders are not limited to the severity of (hypo)manic syndromes but include patterns of clinical course and family history. Caution in the use of potentially mood-destabilizing agents is warranted during the early course of bipolar II disorder.

**Supplementary Information:**

The online version contains supplementary material available at 10.1186/s40345-023-00304-9.

## Background

Since almost thirty years, bipolar II disorder has received an official recognition in the Diagnostic and Statistical Manual of Mental Disorders (DSM-IV) (American Psychiatric Association 1994). Its definition has been based on the concept of hypomania, a term originally coined by the German neuropsychiatrist Emanuel Mendel at the end of the 19th century (Mendel, 1881), and currently integrated in official psychiatric nosography to describe milder, non-psychotic and less impairing, but not qualitatively different, forms of mania (American Psychiatric Association 1994). Most of early research has supported differentiation of bipolar II disorder from major depressive disorder and its inclusion within the bipolar spectrum (e.g. Coryell et al. 1989, 1984; Endicott et al. 1985; Kupfer et al. 1988).

Only in the last two decades, several studies focused on clinical differences between bipolar I and II disorders. While some authors favored a dichotomous model of (hypo)manic syndromes (Parker et al. 2016, 2020), others questioned the validity of the bipolar I-II dichotomy based on the lack of clear biological borders between the two conditions, the arbitrariness of diagnostic thresholds, and the utility of dimensional models of bipolar disorder (Gitlin and Malhi 2020). They argued that combining bipolar I and II disorders into a single category could lead to more consistent clinical management and promote the concept of bipolar spectrum accommodating different clinical expressions of bipolarity (e.g., mixed states) (Gitlin and Malhi 2020). Nevertheless, it could be argued that merging bipolar disorder types does not resolve the issue of biological heterogeneity nor that of diagnostic borders with other nosographic categories, and may, on the contrary, reduce the attention to subtle forms of bipolarity. Moreover, implementing dimensional models does not require abandoning previous knowledge based on categorical distinctions. Finally, as several authors underlined, the diagnosis of bipolar II disorder has important treatment implications, with respect to bipolar I disorder on one hand and major depressive disorder on the other hand (Parker 2021; Post 2019; Vieta 2019).

In this study, we examined sociodemographic and clinical differences between patients diagnosed with bipolar I and II disorders. An emphasis was given to the early phases of the disorders, conventionally defined here as the period comprising the first five recurrences of the illness. Greater attention to the early course of bipolar disorder is warranted, given the need for early recognition of bipolar disorder and the potential detrimental effects of delayed or inappropriate treatments.

## Methods

### Participants

Four cohorts of patients recruited between April 1994 and March 2022 were gathered from different datasets of patients followed longitudinally, in most instances for 10 or more years. The cohorts were from the Mood Disorders program at the Nova Scotia Health Authority and the Maritime Bipolar Registry (N = 510), from the Mood Disorders Program at the McGill University Health Centre (N = 120), from the affective disorder clinics of the Hamilton Psychiatric Hospital and the Royal Ottawa Hospital (N = 119), and from a sample recruited in Canada, Germany, Czech Republic, Sweden, Denmark, and Austria (N = 59) by the International Group for The Study of Lithium Treated Patients for a previous genetic study (Turecki et al. 1998). All patients provided informed consent.

### Assessment

Overall, 808 subjects diagnosed with bipolar I or bipolar II disorder according to DSM-IV criteria (American Psychiatric Association 1994) were eligible for the analyses. All patients were interviewed using structured or semi-structured interviews (SADS-L, SCID, or DIGS). At the study evaluation, participating psychiatrists completed a detailed assessment covering patients’ sociodemographic variables, diagnosis and clinical features including psychiatric and medical comorbidities, family history of psychiatric disorders, illness course, history of suicidal behavior, response to treatment and treatment adherence. The General Assessment of Functioning (GAF) scale was used to evaluate functional impairments. Whenever possible, family members were also interviewed to corroborate information on family history. Additional variables describing the onset and early course of the illness (i.e., within the first five episodes of illness) were extracted from narrative summaries of all interviews and the long-term follow-up data including clinical charts and medical records. The threshold of five episode was chosen to avoid potential confounding from long-term treatment effects on the clinical course of the disease. When counting the number of lifetime episodes, multiphasic cycles (e.g., mania-depression-interval) and continuous cycling periods were considered as distinct illness episodes, separate from isolated manic and depressive episodes. A free interval of at least 8 weeks was required to consider two episodes as distinct.

Treatment response to lithium salts, valproic acid derivatives and lamotrigine were evaluated in previously exposed patients using the Retrospective Criteria of Long-Term Treatment Response in Research Subjects with Bipolar Disorder, commonly referred to as the “Alda Scale” (Manchia et al. 2013). The scale is composed of two sections: the criterion A section is used to rate illness activity while on treatment based on frequency, severity and duration of episodes, on a scale from 0 (= no change or worsening) to 10 (= complete response, no recurrences, no residual symptoms); the criterion B section is composed of five items used to estimate the reliability of a causal relationship between treatment and response. The total score of the scale is calculated by subtracting the B score from the A score. Patients with total score ≥ 7 were considered full responders.

### Statistical analysis

All analyses were performed using R Statistical Software (Foundation for Statistical Computing, Vienna, Austria). A statistical significance level of p < 0.05 was set for all tests. Descriptive statistics were used to summarize the characteristics of the sample. Univariate comparisons between patients diagnosed with bipolar I vs. bipolar II disorder were conducted using Pearson’s chi-squared tests (or Fisher’s exact test) for categorical variables, and Wilcoxon rank-sum test for continuous variables, after excluding normality based on Shapiro-Wilk test. Pairwise Fisher’s exact test was used for post-hoc comparisons of multinomial variables, using Benjamini–Hochberg procedure as false discovery rate correction method. After pruning for variables with missingness > 20%, 10 variables showing significant differences between groups at the univariate level were selected. Missing data were imputed through k-Nearest Neighbor Imputation using the VIM package in R and the variables were entered as predictors in a multivariate logistic regression model with bipolar disorder type as response variable. Backward stepwise model selection based on minimizing the Akaike Information Criterion (AIC) was finally applied.

## Results

### Sample characteristics

The sample included 808 patients diagnosed with bipolar I (N = 587, 72.7%) or bipolar II disorder (N = 221, 27.4%). Their mean age was 45.64 ± 13.56 years (range: 15–82 years), with more women (59.7%) than men. Most subjects were married (46.6%) or single (29.3%). More than one fourth of the patients were working (20.3% full-time and 8.6% part-time), while another fourth were on disability (26.5%). The remaining patients were on social assistance (12.7%), retired (9.7%), unemployed (8.0%), or students (7.1%). Overall, most patients showed adequate functioning at the time of the assessment, as indicated by three fourths of the sample scoring 60 or above on the GAF scale.

As for bipolar disorder features, the onset of mood disorders was between 17 and 30 years for half of the patients. Mean illness duration was 20.87 ± 12.44 years (range: 1–61 years). The clinical course was completely episodic in 30.6% of patients, while 31.6% of subjects had residual symptoms, and 30.1% had a chronic fluctuating course. Only few subjects had had a single episode (2.2%) or completely chronic course (5.4%). A history of suicide attempts was frequent, with 36.5% of patients having attempted suicide at least once; approximately one third of patients had a first or second degree relative who attempted suicide (32.7%).

Additional information on first episode features and early course characteristics (i.e., pertaining to the first five episodes) were available approximately for three fourths to one half of the patients, depending on the variable selected. Notably, the first episode polarity was most frequently depressive (56.5%), and about a half of the first five episodes were of depressive polarity. Less than a fifth of patients experienced (hypo)mania-depression cycles during their early course (18.7%); depression-(hypo)mania cycles were almost half as frequent (11.4%). Information on psychotic features was available in about a half of the sample. In this subgroup, lifetime psychosis was found in 59.4% of cases; in 23.3% psychosis was present at onset and in 52.4% occurred within the first five episodes.

Further information on the sample characteristics, including psychiatric and medical comorbidities, family history, and treatment response, is summarized in Supplementary Table 1. Data on treatment response were available in approximately two thirds of patients for lithium, in one fifth for valproic acid, and in about one tenth for lamotrigine, based on previous exposure to these medications, sufficient length of exposure (≥ 6 months), adequate doses and/or standard blood levels, and the lack of combination with other mood stabilizers.

### Univariate comparisons

Sociodemographic and clinical differences between the two subtypes of bipolar disorder are summarized in Table 1. Briefly, there was a larger proportion of women in the bipolar II group; bipolar II patients were also older at the assessment. While the age at onset of mood disorders and depressive episodes was similar in the two groups, patients with bipolar II disorder had a significantly later onset of (hypo)manic episodes and first psychiatric treatment.

**Table 1:** Sociodemographic and clinical differences between patients diagnosed with bipolar I disorder (N = 587) and bipolar II disorder (N = 221). Pearson’s chi-squared tests (or Fisher’s exact test [*]) has been used for categorical variables, Wilcoxon rank-sum test for continuous variables. P < 0.05 are shown in bold. Pairwise Fisher’s exact test was used for post-hoc comparisons of multinomial variables, using Benjamini–Hochberg procedure as false discovery rate (FDR) correction method. Pairwise significant differences (FDR-p < 0.05) between individual categories are indicated with lowercase letters. Abbreviations: M = median; IQR = interquartile range; SMD = standardized mean difference

	Bipolar I disorder	Bipolar II disorder		
Sociodemographic variables	**N (%) / M [IQR]**	**N (%) / M [IQR]**	**SMD**	**p**
Age (years)	44.09 [34.80, 53.59]	47.99 [38.75, 58.17]	0.201	**0.005**
Sex (female)	335 (57.1)	147 (66.5)	0.195	**0.018**
Marital status*			0.420	**< 0.001**
Single	137 (33.9)	29 (17.8)		a,b
Married	170 (42.1)	94 (57.7)		a
Divorced	91 (22.5)	34 (20.9)		
Widowed	6 (1.5)	6 (3.7)		b
Socioeconomic status			0.375	**0.024**
Work full-time	86 (22.4)	23 (15.1)		
Work part-time	35 (9.1)	11 (7.2)		
Unemployment insurance	33 (8.6)	10 (6.6)		
Social assistance	48 (12.5)	20 (13.2)		
Disabled	106 (27.6)	36 (23.7)		
Other	21 (5.5)	17 (11.2)		
Retired	29 (7.6)	23 (15.1)		
Student	26 (6.8)	12 (7.9)		
General Assessment of Functioning (GAF) score	70.00 [60.00, 80.00]	70.00 [60.00, 80.00]	0.049	0.828
**Mood disorder features**				
Age at onset of major depression (years)	20.00 [16.00, 26.00]	22.00 [16.00, 28.00]	0.134	0.373
Age at onset of mania or hypomania (years)	25.00 [19.00, 33.00]	28.00 [20.00, 37.00]	0.230	**0.022**
Age at onset of mood disorders (years)	22.00 [17.00, 29.00]	23.00 [18.00, 31.00]	0.095	0.389
Age at first psychiatric treatment (years)	31.00 [23.00, 40.00]	38.00 [28.75, 47.00]	0.437	**< 0.001**
Illness duration (years)	19.00 [11.00, 29.00]	22.00 [12.00, 30.00]	0.146	**0.048**
Illness duration before treatment			0.409	**0.010**
Treatment before mood disorder onset	33 (11.7)	8 (6.7)		
Early treatment (from onset to 2 years after)	87 (31.0)	22 (18.3)		a
Average delay (3 to 9 years from onset)	61 (21.7)	29 (24.2)		
Late treatment (9 to 10 years from onset)	51 (18.1)	26 (21.7)		
Very late treatment (20 or more years from onset)	49 (17.4)	35 (29.2)		a
**Psychiatric comorbidity**				
Social anxiety	90 (20.2)	45 (25.1)	0.119	0.210
Panic disorder	95 (20.5)	43 (24.2)	0.087	0.370
Generalized anxiety	133 (29.9)	61 (33.9)	0.086	0.377
Obsessive compulsive disorder	49 (10.6)	25 (14.2)	0.109	0.258
Substance use disorders	163 (35.2)	47 (26.7)	0.185	0.051
Attention-deficit/hyperactivity disorders	23 (5.3)	10 (5.9)	0.025	0.933
Learning disabilities	21 (4.8)	10 (5.8)	0.043	0.774
Primary insomnia	43 (9.9)	33 (19.1)	0.263	**0.003**
Personality disorders	52 (12.1)	21 (12.7)	0.018	0.958
**Medical history**				
Diabetes mellitus	44 (11.0)	15 (9.0)	0.065	0.586
Hypertension	48 (12.2)	32 (19.4)	0.200	**0.036**
Menstrual abnormalities	61 (30.0)	34 (33.3)	0.071	0.650
Thyroid problems	88 (22.2)	51 (32.3)	0.229	**0.018**
Head injury	96 (28.1)	29 (21.0)	0.165	0.139
Neurological comorbidity	15 (4.7)	7 (5.4)	0.031	0.953
Migraine	59 (15.4)	45 (29.2)	0.335	**< 0.001**
**Family history**				
Bipolar disorder (proportion of first-degree relatives affected)	0.00 [0.00, 0.03]	0.00 [0.00, 0.03]	0.003	0.960
Major depressive disorder (proportion of first-degree relatives affected)	0.00 [0.00, 0.20]	0.12 [0.00, 0.25]	0.189	**0.011**
Schizophrenia (proportion of first-degree relatives affected)	0.00 [0.00, 0.00]	0.00 [0.00, 0.00]	0.057	0.982
Schizoaffective disorder (proportion of first-degree relatives affected)	0.00 [0.00, 0.00]	0.00 [0.00, 0.00]	0.034	0.659
Anxiety disorders (proportion of first-degree relatives affected)	0.00 [0.00, 0.00]	0.00 [0.00, 0.00]	0.083	**0.040**
Other psychiatric disorders (proportion of first-degree relatives affected)	0.14 [0.00, 0.33]	0.20 [0.00, 0.33]	0.142	0.180
First-degree family history of completed suicide	33 (8.1)	12 (7.6)	0.019	0.980
First-degree family history of suicide attempt	83 (20.5)	43 (27.7)	0.169	0.087
First- or second-degree family history of completed suicide	72 (18.0)	24 (15.6)	0.063	0.592
First- or second-degree family history of suicide attempt	129 (32.3)	51 (33.6)	0.026	0.864

There were no significant differences in most comorbid psychiatric disorders. However, among patients with bipolar II disorders there were more individuals with medical comorbid conditions, namely, hypertension, thyroid problems and migraine. The family history of bipolar II patients was characterized by a higher proportion of first-degree relatives affected by major depression and by anxiety disorders compared to family history of patients with bipolar I disorder.

Univariate analyses of the lifetime clinical course are summarized in Table 2. The main difference was in predominant polarity with bipolar II disorder more frequently diagnosed in patients with depressive predominant polarity than in those with balanced or manic predominant polarity. Moreover, bipolar II disorder was more frequently diagnosed in those with a chronic fluctuating course.

**Table 2:** Lifetime clinical course differences between patients diagnosed with bipolar I disorder (N = 587) and bipolar II disorder (N = 221). Pearson’s chi-squared tests (or Fisher’s exact test [*]) has been used for categorical variables, Wilcoxon rank-sum test for continuous variables. P < 0.05 are shown in bold. Pairwise Fisher’s exact test was used for post-hoc comparisons of multinomial variables, using Benjamini–Hochberg procedure as false discovery rate (FDR) correction method. Pairwise significant differences (FDR-p < 0.05) between individual categories are indicated with lowercase letters. Abbreviations: M = median; IQR = interquartile range; SMD = standardized mean difference

	Bipolar I disorder	Bipolar II disorder		
Lifetime clinical course features	**N (%) / M [IQR]**	**N (%) / M [IQR]**	**SMD**	**p**
Clinical course*			0.487	**< 0.001**
Single episode	14 (3.1)	0 (0.0)		a,b
Completely episodic	155 (34.2)	36 (21.1)		c
Episodic with residual symptoms	147 (32.5)	50 (29.2)		d
Chronic fluctuating	115 (25.4)	73 (42.7)		b,c,d
Chronic/Deteriorating/Continuous cycling	22 (4.9)	12 (7.0)		a
Predominant polarity			0.735	**< 0.001**
Manic	156 (28.6)	12 (6.0)		a,b
Balanced	229 (42.0)	75 (37.3)		a,c
Depressive	160 (29.4)	114 (56.7)		b,c
Number of lifetime isolated (hypo)manic episodes			0.383	**< 0.001**
None	53 (9.8)	31 (15.5)		a,b
One episode	114 (21.0)	62 (31.0)		c,d
Two episodes	107 (19.7)	28 (14.0)		a,c
Multiple recurrence (3 to 6 episodes)	157 (29.0)	35 (17.5)		b,d
Highly recurrent (hypo)mania (7 or more episodes)	111 (20.5)	44 (22.0)		
Number of lifetime isolated depressive episodes			0.676	**< 0.001**
None	71 (13.1)	5 (2.5)		a,b,c
One episode	104 (19.2)	18 (9.0)		d,e
Recurrent depression (2 to 3 episodes)	154 (28.4)	41 (20.5)		a,f,g
Multiple recurrence (4 to 7 episodes)	125 (23.0)	67 (33.5)		b,d,f
Highly recurrent depression (8 or more episodes)	89 (16.4)	69 (34.5)		c,e,g
Number of lifetime illness episodes (including multiphasic cycles, mixed episodes, continuous cycling)			0.238	**0.038**
Low number of episodes (1 to 4 episodes)	174 (32.6)	49 (25.3)		a
Average number of episodes (5 to 9 episodes)	195 (36.5)	69 (35.6)		
High number of episodes (10 to 15 episodes)	86 (16.1)	31 (16.0)		
Very high number of episodes (16 or more episodes)	79 (14.8)	45 (23.2)		a
Frequency of episodes			0.360	**0.001**
Low recurrence (at most every 4 years)	134 (25.0)	45 (23.1)		a
Average recurrence (at most every 2 years)	155 (28.9)	51 (26.2)		b
High recurrence (at most every year)	101 (18.8)	25 (12.8)		c
Very high recurrence (more than 1 episode per year)	57 (10.6)	14 (7.2)		d
Rapid cycling course (more than 4 episodes per year)	89 (16.6)	60 (30.8)		a,b,c,d
Lifetime history of rapid cycling	89 (25.1)	60 (46.5)	0.457	**< 0.001**
Lifetime history of multiphasic cycles	183 (36.0)	48 (26.7)	0.201	**0.030**
Lifetime history of psychosis			1.389	**< 0.001**
No history of psychosis	79 (26.1)	88 (81.5)		a,b
Mood-congruent psychosis	172 (56.8)	10 (9.3)		a,c
Mood-incongruent/outside episodes	52 (17.2)	10 (9.3)		b,c
Lifetime history of suicide attempts			0.045	0.881
No history of suicide attempts	282 (63.9)	110 (61.8)		
Single attempt	93 (21.1)	40 (22.5)		
Multiple attempts	66 (15.0)	28 (15.7)		

The differences in total numbers of lifetime episodes, including multiphasic cycles, mixed episodes and continuous cycling, were limited to a greater proportion of patients with very high number of episodes (16 or more) in the bipolar II group compared to those with a low number of episodes (1 to 4). Similarly, all the differences in the frequency of episodes were driven by a higher rate of bipolar II disorder in patients with lifetime rapid cycling that itself was more frequent in patients with bipolar II than in those with bipolar I disorder. On the other hand, multiphasic cycles had been more frequently experienced by patients with bipolar I disorder.

Interestingly, among patients with lifetime psychosis, those few with bipolar II disorder showed more frequently mood-incongruent features or psychosis outside of mood episodes than patients with bipolar I disorder. Notably, no significant differences in lifetime suicide attempts nor in family history of suicide was observed between the groups.

Details of the early clinical course are summarized in Table 3. The early course patterns largely mirror the lifetime course of the illness. In patients with bipolar II disorder, the first episode less frequently consisted of (hypo)mania or a (hypo)mania-depression cycle than minor depression, major depression or a depression-(hypo)mania cycle. Psychotic features were significantly more frequent at the onset of bipolar I disorder; patients with bipolar I disorder were more frequently treated from their first episode, either as inpatients or outpatients.

**Table 3:** Early course differences between patients diagnosed with bipolar I disorder (N = 587) and bipolar II disorder (N = 221). Pearson’s chi-squared tests (or Fisher’s exact test [*]) has been used for categorical variables, Wilcoxon rank-sum test for continuous variables. P < 0.05 are shown in bold. Pairwise Fisher’s exact test was used for post-hoc comparisons of multinomial variables, using Benjamini–Hochberg procedure as false discovery rate (FDR) correction method. Pairwise significant differences (FDR-p < 0.05) between individual categories are indicated with lowercase letters. Abbreviations: DM = depressive-(hypo)manic; M = median; MD = (hypo)manic-depressive; IQR = interquartile range; SMD = standardized mean difference

	Bipolar I disorder	Bipolar II disorder		
First episode features	**N (%) / M [IQR]**	**N (%) / M [IQR]**	**SMD**	**p**
Episode duration (weeks)	8.00 [4.00, 20.00]	12.00 [4.00, 24.50]	0.160	0.083
Episode polarity*			0.697	**< 0.001**
(Hypo)mania	143 (33.6)	20 (13.6)		a,b,c
MD biphasic cycle	31 (7.3)	0 (0.0)		d,e,f
Major depression	217 (50.9)	107 (72.8)		a,d
Minor depression	19 (4.5)	11 (7.5)		b,e
DM biphasic cycle	8 (1.9)	7 (4.8)		c,f
Mixed state/Rapid cycling	8 (1.9)	2 (1.4)		
Psychotic features	85 (31.6)	3 (2.8)	0.828	**< 0.001**
Complete remission	266 (76.0)	100 (77.5)	0.036	0.821
Treatment setting			0.616	**< 0.001**
Not treated	117 (31.8)	69 (53.1)		a,b
Outpatient	84 (22.8)	37 (28.5)		a
Inpatient	167 (45.4)	24 (18.5)		b
**Early course (up to the fifth episode)**				
First cycle length (time from onset to first recurrence)			0.367	**0.020**
8 to 48 weeks	75 (21.6)	14 (12.4)		a
52 to 100 weeks	74 (21.3)	21 (18.6)		
104 to 204 weeks	71 (20.4)	21 (18.6)		
209 to 416 weeks	65 (18.7)	21 (18.6)		
417 or more weeks	63 (18.1)	36 (31.9)		a
Average episode duration	8.00 [4.60, 14.00]	10.50 [5.00, 19.95]	0.291	**0.015**
History of early hospitalization	288 (80.0)	54 (46.6)	0.740	**< 0.001**
History of incomplete remission	145 (42.2)	52 (44.4)	0.046	0.745
Proportion of (hypo)manic episodes (%)	0.40 [0.20, 0.60]	0.20 [0.00, 0.40]	0.673	**< 0.001**
Proportion of depressive episodes (%)	0.40 [0.20, 0.60]	0.67 [0.50, 0.80]	0.895	**< 0.001**
Proportion of other episodes (%)	0.00 [0.00, 0.31]	0.00 [0.00, 0.20]	0.270	**0.025**
(Hypo)manic-depressive cycles	94 (23.4)	6 (4.5)	0.566	**< 0.001**
Depressive-(hypo)manic cycles	45 (11.2)	16 (12.0)	0.026	0.916
Rapid cycling	29 (7.2)	22 (16.5)	0.291	**0.003**
Psychotic features	183 (67.5)	12 (11.9)	1.383	**< 0.001**
Antidepressant treatment	148 (57.1)	80 (79.2)	0.488	**< 0.001**
Antipsychotic treatment	142 (54.8)	22 (21.8)	0.723	**< 0.001**
Benzodiazepine treatment	101 (39.0)	31 (30.7)	0.175	0.178
Psychotherapy	30 (11.6)	9 (8.9)	0.088	0.586
Electroconvulsive therapy	30 (11.6)	9 (8.9)	0.088	0.586
Mood stabilizer treatment	197 (76.1)	62 (61.4)	0.321	**0.008**
History of early treatment*	251 (96.9)	93 (92.1)	0.213	0.082
First episode of (hypo)manic or mixed polarity			0.678	**< 0.001**
First episode	190 (44.6)	29 (19.7)		a,b,c
Second episode	127 (29.8)	38 (25.9)		a,d,e
Third or fourth episode	68 (16.0)	44 (29.9)		b,d
Fifth episode or more	41 (9.6)	36 (24.5)		c,e

During the early course of disease, patients with bipolar I disorder had a higher proportion of (hypo)manic episodes as well as multiphasic cycles, mixed episodes and continuous cycling, and a lower proportion of depressive episodes. Importantly, (hypo)mania-depression cycles occurred significantly more often during the early course of bipolar I disorder than in bipolar II disorder, while there was no difference for depression-(hypo)mania cycles. Symptoms of psychosis were also more frequent during the early course of disease in patients with bipolar I disorder while rapid cycling was more frequently observed in the bipolar II group. Patients with bipolar I disorder had been more frequently treated with mood stabilizers and antipsychotics within the first five episodes, while patients with bipolar II disorder more frequently received antidepressant treatments. Importantly, the first episode qualifying the mood disorder as bipolar, that is (hypo)mania, multiphasic cycles, mixed episodes, or continuous cycling, was the first or the second more frequently than all the subsequent ones in patients with bipolar I disorder compared to those with bipolar II, who more often presented a later “bipolar” presentation.

The analyses of treatment response are summarized in Table 4. While lithium response was similar in the two groups, both based on the A score criterion and the total score of the Alda scale, patients with bipolar II disorders differed from the others for the number of episodes before/off treatment (item B1) and for the use of additional medication during the period(s) of stability (item B5). In those with bipolar II disorder, prolonged or systematic use of antidepressants or antipsychotics was more common than the use of low dose of antidepressants or antipsychotics, or the prolonged use of sleep medications. Nevertheless, more than one fourth in each groups required additional medication other than infrequent sleep medications.

**Table 4:** Treatment response differences between patients diagnosed with bipolar I disorder (N = 587) and bipolar II disorder (N = 221). Pearson’s chi-squared tests (or Fisher’s exact test [*]) has been used for categorical variables, Wilcoxon rank-sum test for continuous variables. P < 0.05 are shown in bold. Pairwise Fisher’s exact test was used for post-hoc comparisons of multinomial variables, using Benjamini–Hochberg procedure as false discovery rate (FDR) correction method. Pairwise significant differences (FDR-p < 0.05) between individual categories are indicated with lowercase letters. Abbreviations: M = median; IQR = interquartile range; SMD = standardized mean difference

	Bipolar I disorder	Bipolar II disorder		
Lithium treatment response	**N (%) / M [IQR]**	**N (%) / M [IQR]**	**SMD**	**p**
Lithium responders	157 (37.2)	53 (35.1)	0.044	0.717
A score (improvement)	8.00 [4.00, 9.00]	6.50 [3.25, 9.00]	0.161	0.153
B1 score: number of episodes before/off treatment			0.296	**0.019**
4 or more episodes	291 (74.8)	124 (85.5)		
2 or 3 episodes	67 (17.2)	17 (11.7)		
1 episode	31 (8.0)	4 (2.8)		
B2 score: frequency of episodes before/off treatment			0.215	0.128
Average to high, including rapid cycling	296 (76.1)	119 (82.1)		
Low, spontaneous remissions of 3 or more years	65 (16.7)	22 (15.2)		
1 episode only, risk not known	28 (7.2)	4 (2.8)		
B3 score: duration of the treatment			0.169	0.191
2 or more years	336 (86.4)	117 (80.7)		
1–2 years	33 (8.5)	15 (10.3)		
Less than 1 year	20 (5.1)	13 (9.0)		
B4 score: compliance during period(s) of stability			0.268	0.062
Excellent, e.g., documented by drug levels	342 (88.1)	132 (91.7)		
Good, more than 80% levels in the therapeutic range	26 (6.7)	11 (7.6)		
Poor, repeatedly off treatment	20 (5.2)	1 (0.7)		
B5 score: use of additional medication during the period(s) of stability			0.263	**0.032**
None except infrequent sleep medications	116 (29.9)	41 (28.3)		
Low-dose antidepressants or antipsychotics, or prolonged use of sleep medications	101 (26.0)	24 (16.6)		a
Prolonged or systematic use of antidepressants or antipsychotics	171 (44.1)	80 (55.2)		a
Total score	5.00 [1.00, 8.00]	4.00 [1.00, 8.00]	0.071	0.522
**Valproic acid treatment response**				
Valproic acid responders*	18 (14.0)	2 (4.9)	0.315	0.165
A score (improvement)	7.00 [4.00, 8.00]	5.00 [1.00, 5.75]	0.738	**< 0.001**
B1 score: number of episodes before/off treatment*			0.256	0.495
4 or more episodes	99 (77.3)	36 (85.7)		
2 or 3 episodes	20 (15.6)	5 (11.9)		
1 episode	9 (7.0)	1 (2.4)		
B2 score: frequency of episodes before/off treatment*			0.433	0.085
Average to high, including rapid cycling	91 (71.1)	37 (88.1)		
Low, spontaneous remissions of 3 or more years	28 (21.9)	4 (9.5)		
1 episode only, risk not known	9 (7.0)	1 (2.4)		
B3 score: duration of the treatment			0.470	**0.018**
2 or more years	90 (70.3)	23 (56.1)		a
1–2 years	24 (18.8)	6 (14.6)		
Less than 1 year	14 (10.9)	12 (29.3)		a
B4 score: compliance during period(s) of stability*			0.503	0.063
Excellent, e.g., documented by drug levels	106 (83.5)	40 (97.6)		
Good, more than 80% levels in the therapeutic range	16 (12.6)	1 (2.4)		
Poor, repeatedly off treatment	5 (3.9)	0 (0.0)		
B5 score: use of additional medication during the period(s) of stability*			0.507	**0.043**
None except infrequent sleep medications	14 (10.9)	2 (4.9)		
Low-dose antidepressants or antipsychotics, or prolonged use of sleep medications	23 (18.0)	2 (4.9)		
Prolonged or systematic use of antidepressants or antipsychotics	91 (71.1)	37 (90.2)		
Total score	3.00 [1.00, 5.00]	0.50 [0.00, 3.75]	0.778	**< 0.001**
**Lamotrigine treatment response**				
Lamotrigine responders*	8 (15.7)	3 (9.4)	0.192	0.517
A score (improvement)	5.00 [3.25, 8.00]	6.00 [3.00, 7.75]	0.037	0.880
B1 score: 2 or 3 episodes before/off treatment (vs. 4 or more)*	6 (11.8)	2 (5.9)	0.209	0.467
B2 score: low frequency of episodes (vs. average to high)*	5 (9.8)	2 (5.9)	0.146	0.697
B3 score: duration of the treatment*			0.245	0.550
2 or more years	39 (76.5)	23 (67.6)		
1–2 years	6 (11.8)	7 (20.6)		
Less than 1 year	6 (11.8)	4 (11.8)		
B4 score: compliance during period(s) of stability*			0.091	1.000
Excellent, e.g., documented by drug levels	49 (96.1)	32 (94.1)		
Good, more than 80% levels in the therapeutic range	1 (2.0)	1 (2.9)		
Poor, repeatedly off treatment	1 (2.0)	1 (2.9)		
B5 score: use of additional medication during the period(s) of stability*			0.223	0.589
None except infrequent sleep medications	1 (2.0)	2 (5.9)		
Low-dose antidepressants or antipsychotics, or prolonged use of sleep medications	2 (4.0)	2 (5.9)		
Prolonged or systematic use of antidepressants or antipsychotics	47 (94.0)	30 (88.2)		
Total score	3.50 [1.00, 5.00]	4.00 [0.00, 5.00]	0.067	0.648

Valproic acid-related improvement differed between the groups, with a lower response in patients with bipolar II disorder, both based on the criterion A score and based on the total score of Alda scale. Importantly, compared to patients with bipolar I disorder, those diagnosed with bipolar II had been treated with valproic acid for less than a year more frequently than for 2 or more years, and a trend toward a higher prolonged or systematic use of antidepressants or antipsychotics compared to the use of low dose of antidepressants or antipsychotics, was observed in patients with bipolar II disorder compared to the others. No significant differences between the groups were observed for lamotrigine response.

### Multivariate analysis

After removing variables with missingness > 20%, 10 features showing significant differences between groups at the univariate level remained in the dataset, namely age, sex, age at onset of (hypo)mania, illness duration, predominant polarity, number of lifetime isolated (hypo)manic episodes, number of lifetime isolated depressive episodes, number of lifetime illness episodes (including multiphasic cycles, mixed episodes, continuous cycling), frequency of episodes, and lifetime history of multiphasic cycles. Missingness varied from 14.7% for lifetime history of multiphasic cycles, to 0% for age and sex. After imputation of missing data, all the features were entered in a logistic regression model with bipolar disorder type as the dependent variable (Table 5A). Median categories were chosen as reference categories for all multinomial variables, except for frequency of episodes, where rapid cycling was preferred based on univariate analyses.

According to the full multivariate model, the number of lifetime isolated (hypo)manic and depressive episodes, and the frequency of episodes, were associated with bipolar disorder type. Particularly, patients with bipolar II disorder had significantly more frequently one isolated hypomanic episode rather than two, showed more frequently multiple depressive episodes (4 to 7) or a highly recurrent depression (8 or more episodes) rather than two or three depressive episodes, and more frequently had a history of rapid cycling rather than a very high recurrence of episodes (more than one episode per year). After backward stepwise model selection, five predictors were retained, namely the age at onset of (hypo)mania, the number of lifetime isolated (hypo)manic and depressive episodes, the frequency of episodes, and lifetime history of multiphasic cycles (Table 5B). Similar to the full multivariate model, patients with bipolar II disorder had significantly more frequently none or one isolated hypomanic episode rather than two, showed significantly more often multiple depressive episodes (4 to 7) or a highly recurrent depression (8 or more episodes) rather than two or three depressive episodes, and less frequently had no history of isolated major depressive episodes. Also, they had more frequently a history of rapid cycling rather than very high recurrence (more than one episode per year). Moreover, a higher age at onset of (hypo)mania was positively associated with bipolar II disorder, while a history of lifetime multiphasic cycles was positively associated with bipolar I disorder.

**Table 5:** Logistic regression models predicting bipolar II disorder. P < 0.05 are shown in bold. Abbreviations: AIC = Akaike Information Criterion; CI = confidence interval; OR = odds ratio; ref = reference category

	A. Full multivariate model (AIC = 871.39)			B. Stepwise selected model (AIC = 862.13)		
Variables	**Estimate**	**OR (95% CI)**	**p**	**Estimate**	**OR (95% CI)**	**p**
**(Intercept)**	-2.165	0.11 (0.04–0.32)	**< 0.001**	-2.001	0.14 (0.06–0.31)	**< 0.001**
**Age (years)**	0.012	1.01 (0.98–1.04)	0.387	-	-	-
**Sex [ref = female]**	-0.159	0.85 (0.59–1.22)	0.387	-	-	-
**Age at onset of mania or hypomania (years)**	0.012	1.01 (0.99–1.04)	0.367	0.020	1.02 (1.00-1.04)	**0.012**
**Illness duration (years)**	-0.009	0.99 (0.96–1.02)	0.553	-	-	-
**Predominant polarity [ref = balanced]**						
Depressive	0.396	1.49 (0.89–2.49)	0.133	-	-	-
Manic	-0.543	0.58 (0.26–1.31)	0.189	-	-	-
**Number of lifetime isolated (hypo)manic episodes [ref = two episodes]**						
None	0.669	1.95 (0.94–4.07)	0.074	1.017	2.76 (1.41–5.43)	**0.003**
One episode	0.604	1.83 (1.01–3.31)	**0.046**	0.81	2.25 (1.29–3.91)	**0.004**
Multiple recurrence (3 to 6 episodes)	-0.124	0.88 (0.48–1.63)	0.690	-0.296	0.74 (0.42–1.32)	0.309
Highly recurrent (hypo)mania (7 or more)	0.381	1.46 (0.64–3.35)	0.367	-0.134	0.87 (0.46–1.67)	0.683
**Number of lifetime isolated depressive episodes [ref = recurrent depression (2 to 3 episodes)]**						
None	-0.687	0.50 (0.15–1.65)	0.256	-1.207	0.30 (0.11–0.81)	**0.017**
One episode	-0.285	0.75 (0.37–1.53)	0.433	-0.477	0.62 (0.33–1.17)	0.138
Multiple recurrence (4 to 7 episodes)	0.928	2.53 (1.35–4.75)	**0.004**	0.962	2.62 (1.61–4.24)	**< 0.001**
Highly recurrent depression (8 or more episodes)	1.515	4.55 (1.91–10.86)	**0.001**	1.448	4.25 (2.38–7.59)	**< 0.001**
**Number of lifetime illness episodes (including multiphasic cycles, mixed episodes, continuous cycling) [ref = average number of episodes (5 to 9 episodes)]**						
Low number of episodes (1 to 4 episodes)	0.181	1.20 (0.61–2.35)	0.600	-	-	-
High number of episodes (10 to 15 episodes)	-0.256	0.77 (0.41–1.47)	0.433	-	-	-
Very high number of episodes (16 or more)	-0.518	0.60 (0.22–1.60)	0.304	-	-	-
**Frequency of episodes [ref = rapid cycling (more than 4 episodes per year)]**						
Low recurrence (at most every 4 years)	-0.119	0.89 (0.44–1.80)	0.742	0.001	1 (0.55–1.83)	0.997
Average recurrence (at most every 2 years)	-0.204	0.82 (0.45–1.46)	0.493	-0.131	0.88 (0.51–1.5)	0.631
High recurrence (at most every year)	-0.579	0.56 (0.31–1.03)	0.061	-0.52	0.59 (0.34–1.05)	0.072
Very high recurrence (more than 1 episode/year)	-0.806	0.45 (0.22–0.92)	**0.028**	-0.798	0.45 (0.22–0.91)	**0.026**
**Lifetime history of multiphasic cycles [ref = absent]**	-0.376	0.69 (0.45–1.04)	0.076	-0.445	0.64 (0.43–0.95)	**0.027**

## Discussion

Patients with bipolar II disorder were described by Dunner et al. (1976) as a milder form of bipolar disorder but with a distinct risk of suicide. This ambiguity continues vexing clinicians and researchers to this day; see Gitlin and Malhi (2020) for more details.

In this study we recruited 808 patients with bipolar I or bipolar II disorders from specialized outpatient clinics and compared the two groups on a number of sociodemographic and clinical variables, including lifetime and early course of illness features, as well as treatment response to major mood stabilizers.

We observed similar demographic differences between the two bipolar disorder types as several other authors. Bipolar II patients were more frequently women (Karanti et al. 2020; Pallaskorpi et al. 2015; Vieta et al. 1997; Vinberg et al. 2017), older (Tondo et al. 2022; Vieta et al. 1997), and more often married or widowed (Bega et al. 2012; Dell’Osso et al. 2017a; Tondo et al. 2022; Vieta et al. 1997).

The two groups were comparable with respect to their overall functioning indexed by the GAF score as described previously (Beunders et al. 2022; Judd et al. 2005; Karanti et al. 2020; Rosa et al. 2010). There were no significant differences in lifetime suicide attempts nor in family history of suicide, consistent with a meta-analysis of retrospective studies showing similar rates of suicide attempts in patients with bipolar I and II disorders (Novick et al. 2010). Notably, in the literature, the lethality of suicide attempts has been reported as higher in patients with bipolar II disorder (Tondo et al. 2007; Vieta et al. 1997). A higher proportion of first-degree relatives of bipolar II patients were affected by major depression and by anxiety disorders as reported earlier (Baek et al. 2011; Coryell et al. 1984; Endicott et al. 1985). This is congruent with recent molecular genetic studies that found an increased polygenic risk for major depressive disorder in patients with bipolar II disorder, while the polygenic risk for schizophrenia was higher in bipolar I disorder (Almeida et al. 2020; Coleman et al., 2020; Guzman-Parra et al. 2021; Song et al. 2018; Stahl et al., 2019).

The treatment trajectories of the two groups of patients differed. In particular, we noted a longer delay before proper treatment of bipolar II disorder. As in previous studies (Baldessarini et al. 1999, 2014; Dell’Osso et al. 2017a; Ghaemi et al. 2000; Karanti et al. 2020; Keramatian et al. 2022; Tondo et al. 2022), patients with bipolar II disorder were older at first psychiatric treatment and were referred less frequently after a single episode. They experienced more episodes before receiving lithium treatment and more often had a long delay before any psychiatric treatment (≥ 20 years from onset), rather than treatment at onset or within 2 years from onset. Taken together, these findings suggest more insidious onset of bipolar II disorder. Consistent with this interpretation, in patients with bipolar I disorder, the first “bipolar” episode (i.e., (hypo)mania, multiphasic cycles, mixed episodes, or continuous cycling) was more often the first or the second, while those with bipolar II disorder had more frequently a later “bipolar” presentation and, on average, a later onset of (hypo)manic episodes. More bipolar I disorder patients had a short duration of their first illness cycle, implying a greater need for early stabilization.

With respect to the clinical course, the main focus of our report, we noted multiple differences. While both groups had their first depressive episode at similar age, bipolar II patients had later onset hypomania. More bipolar II patients had depression as their first episode, higher proportion of depressive episodes during the early course, multiple depressive recurrences and depressive predominant polarity. They experienced less often (hypo)mania or a (hypo)mania-depression cycle at onset, had a lower proportion of episodes including manic polarity during their early course, had less frequently (hypo)mania-depression cycles within the first five episodes, showed less often isolated (hypo)manic episodes and multiphasic cycles, and were less often characterized by manic predominant polarity. Although lower (hypo)manic morbidity in bipolar II disorder has been repeatedly described (Baek et al. 2019; Dell’Osso et al. 2017a; Forte et al. 2015; Pallaskorpi et al. 2019; Tondo et al. 2022), it should be noted that hypomania is difficult to recognize both for patients and clinicians, especially during the early course of illness, while the diagnosis and recall of mania is less likely to be missed (Benazzi and Akiskal 2009). Bipolar II patients may mistake hypomania for depression, especially when it is characterized by irritable mood and mixed features (Akiskal et al. 2003; Benazzi 2004). Importantly, in our sample bipolar I and II disorders were equally represented within patients with highly recurrent (hypo)mania. This suggests a possible cluster of bipolar II patients, approximately one fifth, with (hypo)manic morbidity comparable to that of more recurrent forms of bipolar I disorder. Taken together, these results agree with studies showing increased depressive morbidity in patients with bipolar II disorders (Baek et al. 2011, 2019; Belizario et al. 2019; Dell’Osso et al. 2017a, b; Forte et al. 2015; Judd et al. 2003; Pallaskorpi et al. 2019; Tondo et al. 2022; Vieta et al. 1997).

Previous studies also consistently observed a significant association between bipolar II disorder and rapid cycling course or increased cyclicity (Aedo et al. 2018; Baek et al. 2019; Carvalho et al. 2014; Kupka et al. 2003; Tondo et al. 2022). While treatment-emergent (hypo)mania seems to be more frequent and more severe in patients with bipolar I disorder (Bond et al. 2008; Pacchiarotti et al. 2013), bipolar II disorder, as well as younger age at first exposure, may be associated with antidepressants-induced cycle acceleration (Altshuler et al. 1995). Yet, at the same time, several studies found serotonin reuptake inhibitors as a safe short- and long-term treatment option in patients with bipolar II disorder (Amsterdam et al. 2010, 2015, 2016; Amsterdam and Shults 2010; Liu et al. 2017), even in those with rapid cycling course (Amsterdam et al. 2017). Naturalistic studies also reported frequent use of antidepressants in bipolar II disorder (Dell’Osso et al. 2020; Grande et al., 2013; Shinozaki et al. 2022; Tondo et al. 2013). The question of cycle acceleration in relation to antidepressant treatment may be best answered in a prospective observation. Both bipolar II-related features, such as depression-(hypo)mania cycles and atypical depressive symptoms, and unrelated features, such as a younger age at onset, have been associated with rapid cycling course and must be taken into consideration among variables of interest in future studies (Koukopoulos et al. 2013; Valentí et al. 2015).

It is conceivable that at least some of the observed features of the clinical course in our sample might be due to iatrogenic factors. The patients with bipolar II disorder were less frequently treated with mood stabilizers and antipsychotics, and more frequently received antidepressant treatments within their first five episodes, possibly leading to cycle acceleration, rapid cycling and more chronicity. On the other hand, the rates of lithium response were similar in patients with bipolar I and II disorders, consistent with a recent meta-analysis (Hui et al. 2019). It may be that different factors involved in lithium response counteract each other. For example, the occurrence of mania-depression cycles and a shorter pre-treatment duration may favor treatment response in bipolar I disorder, while a history of psychotic symptoms and hospitalizations may be disadvantaging; conversely, bipolar II patients may benefit from a later age at onset, while rapid cycling course or somatic comorbidity reduce the treatment outcome.

While the lack of early treatment, hospitalization and psychosis might imply less severe illness (Chakrabarti and Singh 2022), our results show a greater tendency to chronicity and frequent recurrence in a proportion of patients with bipolar II disorder. The average duration of the first five episodes was significantly longer for bipolar II patients, possibly due to the higher rate of depressive episodes. Moreover, among patients with psychosis, those with bipolar II disorder were more likely to have psychosis with mood-incongruent features and/or psychosis outside mood episodes, also suggesting chronicity (Chakrabarti and Singh 2022; Fennig 1996; Pfennig et al. 2010). Prolonged or systematic use of antidepressant or antipsychotic medications was also more common among patients with bipolar II disorder. Finally, the lifetime clinical course of patients with bipolar II disorder was more frequently rated as chronic fluctuating than episodic.

Whether bipolar I and bipolar II disorders are two distinct disorders or represent two clusters on a continuum of severity of overactive episodes will be difficult to decide based on clinical data alone. A recent study suggested discontinuity with respect to phenomenology of (hypo)mania (Parker et al. 2020) while others argued for a unified diagnosis (Gitlin and Malhi 2020). However, there are no known biological markers to support either side of the dispute. Some family studies cautiously suggested the possibility of bipolar II disorder “breeding true” in families (Simpson et al. 1993; Song et al. 2018). Moreover, the question is complicated by changing diagnostic criteria. Bipolar disorder II was recognized by Research Diagnostic Criteria but was absent in DSM classification until the fourth edition in 1994.

We suggest that bipolar II disorder, in particular, is heterogeneous with some forms closer to bipolar I disorder while others might represent a separate subgroup. To some extent, this speculation is supported by the heterogeneity of the clinical course of bipolar II patients in our study. Bipolar II disorder was more prevalent among patients with the highest number of lifetime episodes compared to those with the lowest. Yet, there were no differences between intermediate categories, suggesting an existence of a subgroup of patients with a highly recurrent bipolar II disorder *versus* a relatively stable bipolar I disorder. This assumption seems to be justified when looking at the frequency of episodes: bipolar II disorder was associated with rapid cycling during the early course of illness and with lifetime history of rapid cycling, as well as with the highest frequency of episodes per year (more than four). Even in this latter case, no differences between lower frequency categories were observed, suggesting that a distinct subgroup of patients with bipolar II disorder may be especially prone to rapid cycling. The contrast between these findings and the excess of bipolar II disorder among patients showing the longest duration of the first cycle of illness, may be explained by a greater heterogeneity of bipolar II disorder, comprising both highly recurrent and more regular course variants.

Based on previous studies, bipolar II disorder is more often characterized by residual mood symptoms and minor depressive fluctuations (Endicott et al. 1985; Judd et al. 2003; Karanti et al. 2020; Rosa et al. 2010; Vinberg et al. 2017). According to some authors, preexisting temperamental affective instability and intense emotional reactivity, i.e., cyclothymia, characterize the course of most patients with bipolar II disorder, determining high rates of subthreshold mood and anxiety symptoms, subtle but chronic functional impairments, and a high frequency of unprovoked episodes (Koukopoulos et al. 2006; Perugi and Akiskal 2002). Temperament and course types have thus been proposed to capture the prognostic heterogeneity of bipolar disorder better than the actual distinction based on the severity of (hypo)manic episodes (Koukopoulos et al. 2006).

The results of our study need to be viewed in the context of its limitations. First, the data came from a retrospective assessment combined with data obtained from long-term follow-up. This could lead to recall bias, especially for the detection of hypomanic episodes, as previously discussed. Furthermore, the diagnostic criteria of bipolar disorder have changed over the years, and even more so for bipolar II disorder. In our study we used DSM-IV criteria as a compromise as the enrollment of patients occurred over a number of years with most participants recruited prior to introduction of DSM-5.

Second, patients were recruited from tertiary-level outpatients setting, and more than two thirds of the sample were diagnosed with bipolar I disorder, resulting in a bipolar I / bipolar II ratio of 2.65. This is similar to some other clinical samples (Dell’Osso et al. 2017a, b), but greater than epidemiological figures from community samples with a typical ratio of 1.0 to 1.5 (Merikangas et al. 2011). The relative excess of bipolar I disorder thus may reflect a bias towards more severe forms of bipolar II disorder compared to other studies (Karanti et al. 2020; Tondo et al. 2022; Vinberg et al. 2017).

Moreover, some of the differences observed may be considered, at least in part, definitional: since psychosis and hospitalization are sufficient to qualify a (hypo)manic episode as manic, their association with bipolar I disorder is to be expected. On the other hand, as the diagnosis of bipolar II disorder requires presence of both depressive and hypomanic episodes while presence of depression is not required for bipolar I diagnosis, one might expect lower depressive morbidity in bipolar I disorder.

## Conclusions

The clinical data alone do not make it possible to state whether the two bipolar disorder types represent biologically distinct categories. However, while not capturing completely the complexity of bipolar disorder phenomenology, course, and longitudinal morbidity, bipolar I and II disorders might define prototypical groups of patients, whose differences are not limited to severity and impairment of (hypo)manic syndromes and whose clinical needs do not fully overlap.

On one hand, bipolar I disorder is characterized by acute onset, episodic course, manic predominant polarity, and psychotic (mood-congruent) features. On the other, bipolar II disorder has more often an insidious onset, a chronic or highly recurrent course, and a high depressive morbidity. Based on our results, the characteristics of the early clinical course are consistent with lifetime course. This is particularly relevant for early detection, differential diagnosis and management considerations. While the two groups had similar functional impairments, rates of suicide attempts and response to lithium, patients with bipolar II disorder experienced a longer delay before proper treatment and were more frequently exposed to antidepressants during the early course of illness. More caution in the use of potentially mood-destabilizing agents is warranted and more studies on predictors of cycle acceleration are needed. As previously suggested (Mitchell et al. 2008; O’Donovan and Alda 2020; Vöhringer and Perlis 2016), a probabilistic approach to the differential diagnosis between major depressive disorder and bipolar disorder may allow timely recognition of bipolarity and initiation of mood stabilization, even for patients with a predominantly depressive course, mild (hypo)manic syndromes and longer cycles at onset. To this aim, further research focusing on potential phenomenological indicators of bipolarity, such as intra-depressive excitatory features (Brancati et al. 2019; Malhi et al. 2016; Sani et al. 2014), as well as on homotypic (Faedda et al. 2015; Taylor et al. 2021) and heterotypic (Brancati et al. 2021; Duffy et al. 2010) precursors of bipolar disorder, is needed.

Finally, given the heterogeneity observed within bipolar disorder types, and especially within bipolar II disorder in terms of (hypo)manic recurrences and rapid cyclicity, additional, not alternative, classifications based on temperament (Koukopoulos et al. 2006; Perugi and Akiskal 2002), cycle sequence (Koukopoulos et al. 2013; Koukopoulos and Ghaemi 2009), and course (Alda 2004; Koukopoulos et al. 2003, 2006) might be considered to provide more carefully delimited phenotypes for pharmacological and biological research.

## Electronic supplementary material

Below is the link to the electronic supplementary material.

Supplementary Material 1

## Data Availability

The datasets used in this paper are available from the corresponding author on reasonable request.
