# Supplementary material for: Differential characteristics of bipolar I and II disorders: a retrospective, cross-sectional evaluation of clinical features, illness course, and response to treatment
Source: Int J Bipolar Disord. 2023 Jul 14;11:25. doi: 10.1186/s40345-023-00304-9 (PMC10349025; doi:10.1186/s40345-023-00304-9)
Supplement: Supplementary file 1 — Supplementary Material 1 [file 40345_2023_304_MOESM1_ESM.docx]

**Supplementary table 1. Characteristics of the sample (N = 808).** Abbreviations: DM = depressive-(hypo)manic; M = median; MD = (hypo)manic-depressive; IQR = interquartile range.

|  | N (%) / M [IQR] | Missing (%) |
| --- | --- | --- |
| Sociodemographic variables |  |  |
| Age (years) | 45.29 [35.53, 54.90] | 0.0 |
| Sex (female) | 482 (59.7) | 0.0 |
| Marital status |  | 29.8 |
| Single | 166 (29.3) |  |
| Married | 264 (46.6) |  |
| Divorced | 125 (22.0) |  |
| Widowed | 12 (2.1) |  |
| Socioeconomic status |  | 33.7 |
| Work full-time | 109 (20.3) |  |
| Work part-time | 46 (8.6) |  |
| Unemployment insurance | 43 (8.0) |  |
| Social assistance | 68 (12.7) |  |
| Disabled | 142 (26.5) |  |
| Other | 38 (7.1) |  |
| Retired | 52 (9.7) |  |
| Student | 38 (7.1) |  |
| General Assessment of Functioning (GAF) score | 70.00 [60.00, 80.00] | 34.4 |
| Mood disorder features |  |  |
| Age at onset of minor depression (years) | 16.50 [13.25, 19.00] | 99.5 |
| Age at onset of major depression (years) | 20.00 [16.00, 27.00] | 64.7 |
| Age at onset of hypomania (years) | 27.00 [19.00, 36.00] | 66.2 |
| Age at onset of mania (years) | 25.50 [20.00, 34.00] | 31.2 |
| Age at onset of mania or hypomania (years) | 25.00 [19.00, 35.00] | 4.8 |
| Age at onset of mood disorders (years) | 22.00 [17.00, 30.00] | 3.7 |
| Age at first psychiatric treatment (years) | 33.00 [24.00, 42.00] | 50.2 |
| Illness duration (years) | 20.00 [11.00, 29.00] | 3.7 |
| Illness duration before treatment |  | 50.4 |
| Treatment before mood disorder onset | 41 (10.2) |  |
| Early treatment (from onset to 2 years after) | 109 (27.2) |  |
| Average delay (3 to 9 years from onset) | 90 (22.4) |  |
| Late treatment (9 to 10 years from onset) | 77 (19.2) |  |
| Very late treatment (20 or more years from onset) | 84 (20.9) |  |
| Clinical course |  | 22.8 |
| Single episode | 14 (2.2) |  |
| Completely episodic | 191 (30.6) |  |
| Episodic with residual symptoms | 197 (31.6) |  |
| Chronic fluctuating | 188 (30.1) |  |
| Chronic/Deteriorating/Continuous cycling | 34 (5.4) |  |
| Predominant polarity |  | 7.7 |
| Manic | 168 (22.5) |  |
| Balanced | 304 (40.8) |  |
| Depressive | 274 (36.7) |  |
| Number of lifetime isolated (hypo)manic episodes |  | 8.2 |
| None | 84 (11.3) |  |
| One episode | 176 (23.7) |  |
| Two episodes | 135 (18.2) |  |
| Multiple recurrence (3 to 6 episodes) | 192 (25.9) |  |
| Highly recurrent (hypo)mania (7 or more episodes) | 155 (20.9) |  |
| Number of lifetime isolated depressive episodes |  | 8.0 |
| None | 76 (10.2) |  |
| One episode | 122 (16.4) |  |
| Recurrent depression (2 to 3 episodes) | 195 (26.2) |  |
| Multiple recurrence (4 to 7 episodes) | 192 (25.8) |  |
| Highly recurrent depression (8 or more episodes) | 158 (21.3) |  |
| Number of lifetime illness episodes (including multiphasic cycles, mixed episodes, continuous cycling) |  | 9.9 |
| Low number of episodes (1 to 4 episodes) | 223 (30.6) |  |
| Average number of episodes (5 to 9 episodes) | 264 (36.3) |  |
| High number of episodes (10 to 15 episodes) | 117 (16.1) |  |
| Very high number of episodes (16 or more episodes) | 124 (17.0) |  |
| Frequency of episodes |  | 9.5 |
| Low recurrence (at most every 4 years) | 179 (24.5) |  |
| Average recurrence (at most every 2 years) | 206 (28.2) |  |
| High recurrence (at most every year) | 126 (17.2) |  |
| Very high recurrence (more than one episode per year) | 71 (9.7) |  |
| History of rapid cycling (countless episodes) | 149 (20.4) |  |
| Lifetime history of rapid cycling | 149 (30.8) | 40.2 |
| Lifetime history of multiphasic cycles | 231 (33.5) | 14.7 |
| Lifetime history of psychosis |  | 49.1 |
| No history of psychosis | 167 (40.6) |  |
| Mood-congruent psychosis | 182 (44.3) |  |
| Mood-incongruent/outside episodes | 62 (15.1) |  |
| Lifetime history of suicide attempts |  | 23.4 |
| No history of suicide attempts | 392 (63.3) |  |
| Single attempt | 133 (21.5) |  |
| Multiple attempts | 94 (15.2) |  |
| First episode features |  |  |
| Episode duration (weeks) | 8.00 [4.00, 20.00] | 52.0 |
| Episode polarity |  | 29.1 |
| (Hypo)mania | 163 (28.5) |  |
| MD biphasic cycle | 31 (5.4) |  |
| Major depression | 324 (56.5) |  |
| Minor depression | 30 (5.2) |  |
| DM biphasic cycle | 15 (2.6) |  |
| Mixed state/Rapid cycling | 10 (1.7) |  |
| Psychotic features | 88 (23.3) | 53.2 |
| Complete remission | 366 (76.4) | 40.7 |
| Treatment setting |  | 38.4 |
| Not treated | 186 (37.3) |  |
| Outpatient | 121 (24.3) |  |
| Inpatient | 191 (38.4) |  |
| Early course (up to the fifth episode) |  |  |
| First cycle length (time from onset to first recurrence) |  | 42.9 |
| 8 to 48 weeks | 89 (19.3) |  |
| 52 to 100 weeks | 95 (20.6) |  |
| 104 to 204 weeks | 92 (20.0) |  |
| 209 to 416 weeks | 86 (18.7) |  |
| 417 or more weeks | 99 (21.5) |  |
| Average episode duration | 8.80 [4.75, 15.60] | 42.5 |
| History of early hospitalization | 342 (71.8) | 41.1 |
| History of incomplete remission | 197 (42.7) | 42.9 |
| Proportion of (hypo)manic episodes (%) | 0.33 [0.20, 0.50] | 33.8 |
| Proportion of depressive episodes (%) | 0.50 [0.25, 0.67] | 33.8 |
| Proportion of other episodes (%) | 0.00 [0.00, 0.25] | 33.8 |
| (Hypo)manic-depressive cycles | 100 (18.7) | 33.8 |
| Depressive-(hypo)manic cycles | 61 (11.4) | 33.8 |
| Rapid cycling | 51 (9.5) | 33.8 |
| Psychotic features | 195 (52.4) | 54.0 |
| Antidepressant treatment | 228 (63.3) | 55.4 |
| Antipsychotic treatment | 164 (45.6) | 55.4 |
| Benzodiazepine treatment | 132 (36.7) | 55.4 |
| Psychotherapy | 39 (10.8) | 55.4 |
| Electroconvulsive therapy | 39 (10.8) | 55.4 |
| Mood stabilizer treatment | 259 (71.9) | 55.4 |
| History of early treatment | 344 (95.6) | 55.4 |
| First episode of (hypo)manic or mixed polarity |  | 29.1 |
| First episode | 219 (38.2) |  |
| Second episode | 165 (28.8) |  |
| Third or fourth episode | 112 (19.5) |  |
| Fifth episode or more | 77 (13.4) |  |
| Psychiatric comorbidity |  |  |
| Social anxiety | 135 (21.6) | 22.6 |
| Panic disorder | 138 (21.5) | 20.7 |
| Generalized anxiety | 194 (31.0) | 22.6 |
| Obsessive compulsive disorder | 74 (11.6) | 21.0 |
| Substance use disorders | 210 (32.9) | 20.9 |
| Attention-deficit/hyperactivity disorders | 33 (5.5) | 25.2 |
| Learning disabilities | 31 (5.1) | 25.0 |
| Primary insomnia | 76 (12.5) | 24.8 |
| Personality disorders | 73 (12.3) | 26.6 |
| Medical history |  |  |
| Diabetes mellitus | 59 (10.4) | 30.0 |
| Hypertension | 80 (14.3) | 30.7 |
| Menstrual abnormalities | 95 (31.1) | 62.3 |
| Thyroid problems | 139 (25.0) | 31.3 |
| Head injury | 125 (26.0) | 40.6 |
| Neurological comorbidity | 22 (4.9) | 44.9 |
| Migraine | 104 (19.4) | 33.7 |
| Family history |  |  |
| Bipolar disorder (proportion of first-degree relatives affected) | 0.00 [0.00, 0.05] | 9.2 |
| Major depressive disorder (proportion of first-degree relatives affected) | 0.06 [0.00, 0.20] | 25.5 |
| Schizophrenia (proportion of first-degree relatives affected) | 0.00 [0.00, 0.00] | 26.1 |
| Schizoaffective disorder (proportion of first-degree relatives affected) | 0.00 [0.00, 0.00] | 10.6 |
| Anxiety disorders (proportion of first-degree relatives affected) | 0.00 [0.00, 0.00] | 26.1 |
| Other psychiatric disorders (proportion of first-degree relatives affected) | 0.17 [0.00, 0.33] | 26.1 |
| First-degree family history of completed suicide | 45 (8.0) | 30.4 |
| First-degree family history of suicide attempt | 126 (22.5) | 30.8 |
| First- or second-degree family history of completed suicide | 96 (17.3) | 31.3 |
| First- or second-degree family history of suicide attempt | 180 (32.7) | 31.8 |
| Lithium treatment response |  |  |
| Lithium responders | 210 (36.6) | 29.1 |
| A score (improvement) | 8.00 [4.00, 9.00] | 33.9 |
| B1 score: number of episodes before/off treatment |  | 33.9 |
| 4 or more episodes | 415 (77.7) |  |
| 2 or 3 episodes | 84 (15.7) |  |
| 1 episode | 35 (6.6) |  |
| B2 score: frequency of episodes before/off treatment |  | 33.9 |
| Average to high, including rapid cycling | 415 (77.7) |  |
| Low, spontaneous remissions of 3 or more years | 87 (16.3) |  |
| 1 episode only, risk not known | 32 (6.0) |  |
| B3 score: duration of the treatment |  | 33.9 |
| 2 or more years | 453 (84.8) |  |
| 1-2 years | 48 (9.0) |  |
| Less than 1 year | 33 (6.2) |  |
| B4 score: compliance during period(s) of stability |  | 34.2 |
| Excellent, e.g., documented by drug levels | 474 (89.1) |  |
| Good, more than 80% levels in the therapeutic range | 37 (7.0) |  |
| Poor, repeatedly off treatment | 21 (3.9) |  |
| B5 score: use of additional medication during the period(s) of stability |  | 34.0 |
| None except infrequent sleep medications | 157 (29.5) |  |
| Low-dose antidepressants or antipsychotics, or prolonged use of sleep medications | 125 (23.5) |  |
| Prolonged or systematic use of antidepressants or antipsychotics | 251 (47.1) |  |
| Total score | 5.00 [1.00, 8.00] | 33.8 |
| Valproic acid treatment response |  |  |
| Valproic acid responders | 20 (11.8) | 79.0 |
| A score (improvement) | 6.00 [3.00, 8.00] | 79.0 |
| B1 score: number of episodes before/off treatment |  | 79.0 |
| 4 or more episodes | 135 (79.4) |  |
| 2 or 3 episodes | 25 (14.7) |  |
| 1 episode | 10 (5.9) |  |
| B2 score: frequency of episodes before/off treatment |  | 79.0 |
| Average to high, including rapid cycling | 128 (75.3) |  |
| Low, spontaneous remissions of 3 or more years | 32 (18.8) |  |
| 1 episode only, risk not known | 10 (5.9) |  |
| B3 score: duration of the treatment |  | 79.1 |
| 2 or more years | 113 (66.9) |  |
| 1-2 years | 30 (17.8) |  |
| Less than 1 year | 26 (15.4) |  |
| B4 score: compliance during period(s) of stability |  | 79.2 |
| Excellent, e.g., documented by drug levels | 146 (86.9) |  |
| Good, more than 80% levels in the therapeutic range | 17 (10.1) |  |
| Poor, repeatedly off treatment | 5 (3.0) |  |
| B5 score: use of additional medication during the period(s) of stability |  | 79.1 |
| None except infrequent sleep medications | 16 (9.5) |  |
| Low-dose antidepressants or antipsychotics, or prolonged use of sleep medications | 25 (14.8) |  |
| Prolonged or systematic use of antidepressants or antipsychotics | 128 (75.7) |  |
| Total score | 3.00 [0.00, 5.00] | 79.0 |
| Lamotrigine treatment response |  |  |
| Lamotrigine responders | 11 (13.3) | 89.7 |
| A score (improvement) | 6.00 [3.00, 8.00] | 89.6 |
| B1 score: 2 or 3 episodes before/off treatment (vs. 4 or more) | 8 (9.4) | 89.5 |
| B2 score: low frequency of episodes (vs. average to high) | 7 (8.2) | 89.5 |
| B3 score: duration of the treatment |  | 89.5 |
| 2 or more years | 62 (72.9) |  |
| 1-2 years | 13 (15.3) |  |
| Less than 1 year | 10 (11.8) |  |
| B4 score: compliance during period(s) of stability |  | 89.5 |
| Excellent, e.g., documented by drug levels | 81 (95.3) |  |
| Good, more than 80% levels in the therapeutic range | 2 (2.4) |  |
| Poor, repeatedly off treatment | 2 (2.4) |  |
| B5 score: use of additional medication during the period(s) of stability |  | 89.6 |
| None except infrequent sleep medications | 3 (3.6) |  |
| Low-dose antidepressants or antipsychotics, or prolonged use of sleep medications | 4 (4.8) |  |
| Prolonged or systematic use of antidepressants or antipsychotics | 77 (91.7) |  |
| Total score | 4.00 [0.75, 5.00] | 89.6 |
